# Cerebral desaturation in heart failure: Potential prognostic value and physiologic basis

**DOI:** 10.1371/journal.pone.0196299

**Published:** 2018-04-24

**Authors:** Yu-Jen Chen, Jong-Shyan Wang, Chih-Chin Hsu, Pyng-Jing Lin, Feng-Chun Tsai, Ming-Shien Wen, Chi-Tai Kuo, Shu-Chun Huang

**Affiliations:** 1 Department of Physical Medicine and Rehabilitation, Chang Gung Memorial Hospital, Linkou, Taiwan; 2 Healthy Aging Research Center, Graduate Institute of Rehabilitation Science, Medical College, Chang Gung University, Taoyuan, Taiwan; 3 Department of Physical Medicine and Rehabilitation, Chang Gung Memorial Hospital, Keelung, Taiwan; 4 Division of Thoracic and Cardiovascular Surgery, Chang Gung Memorial Hospital, Linkou, Taiwan; 5 Cardiovascular Division, Chang Gung Memorial Hospital, Chang Gung University College of Medicine, Taoyuan, Taiwan; Freeman Hospital, UNITED KINGDOM

## Abstract

Cerebral tissue oxygen saturation (SctO_2_) reflects cerebral perfusion and tissue oxygen consumption, which decline in some patients with heart failure with reduced ejection fraction (HFrEF) or stroke, especially during exercise. Its physiologic basis and clinical significance remain unclear. We aimed to investigate the association of SctO_2_ with oxygen transport physiology and known prognostic factors during both rest and exercise in patients with HFrEF or stroke. Thirty-four HFrEF patients, 26 stroke patients, and 17 healthy controls performed an incremental cardiopulmonary exercise test using a bicycle ergometer. Integrated near-infrared spectroscopy and automatic gas analysis were used to measure cerebral tissue oxygenation and cardiac and ventilatory parameters. We found that SctO_2_ (rest; peak) were significantly lower in the HFrEF (66.3±13.3%; 63.4±13.8%,) than in the stroke (72.1±4.2%; 72.7±4.5%) and control (73.1±2.8%; 72±3.2%) groups. In the HFrEF group, SctO_2_ at rest (SctO_2rest_) and peak SctO_2_ (SctO_2peak_) were linearly correlated with brain natriuretic peptide (BNP), peak oxygen consumption ($\dot{V}O_{2peak}$), and oxygen uptake efficiency slope (*r* between -0.561 and 0.677, *p* < 0.001). Stepwise linear regression showed that SctO_2rest_ was determined by partial pressure of end-tidal carbon dioxide at rest (P_ET_CO_2rest_), hemoglobin, and mean arterial pressure at rest (MAP_rest_) (adjusted R = 0.681, *p* < 0.05), while SctO_2peak_ was mainly affected by peak carbon dioxide production ($\dot{V}CO_{2peak}$) (adjusted R = 0.653, *p* < 0.05) in patients with HFrEF. In conclusion, the study delineates the relationship of cerebral saturation and parameters associated with oxygen delivery. Moreover, SctO_2peak_ and SctO_2rest_ are correlated with some well-recognized prognostic factors in HFrEF, suggesting its potential prognostic value.

## Introduction

Cerebral desaturation may occur in patients with heart failure with reduced ejection fraction (HFrEF) or stroke [1, 2]. The former is due to insufficient cardiac output and dead space ventilation. The latter could be due to disruption of cerebral blood flow (CBF) from vascular abnormalities and neurologic deficit-related respiratory muscle weakness [3–5]. The brain utilizes 20% of the total oxygen consumption at rest [6]. Cerebral oxygenation is determined by arterial oxygen concentration, CBF, and cerebral tissue oxygen consumption, which represent oxygen supply and demand [7]. Insufficient hemoglobin (Hb) concentration, oxygen desaturation, and disruption of cerebral perfusion may result in decreased oxygen supply and thus, cerebral desaturation.

There is evidence that cerebral tissue deoxygenation may limit exercise performance in healthy people. Nielsen et al. demonstrated that when compared with 20% oxygen, 30% inspired oxygen concentration increased exercise performance by decreasing the extent of cerebral desaturation during strenuous exercise; meanwhile, muscle oxygenation was not changed [8]. This study concluded that an elevated inspiratory oxygen fraction increases exercise performance by maintaining cerebral oxygenation rather than through any effect on the working muscles [8]. It is likely that diminished cerebral perfusion may also limit exercise performance in patients with HFrEF. Our previous study revealed that cerebral hypoperfusion is associated with hyperventilation and diminished aerobic capacity in patients with HFrEF [9]. Additionally, frontal cortex dysfunction may impair executive function, resulting in central inhibition [1]. Conversely, poor physical fitness in patients with HFrEF was found to be associated with pathologic change of brain structure, including decreased grey matter volume and cortical thickness, while patients with good physical fitness preserve cerebral structure [10]. Therefore, it is a reasonable conjecture that cerebral tissue oxygen saturation (SctO_2_) in the frontal lobe has a close relationship with exercise performance in patients with HFrEF or stroke, which has not been explored yet.

To go further, peak oxygen consumption ($\dot{V}O_{2peak}$) is a well-recognized significant prognostic factor in patients with HFrEF [11]. If SctO_2_ is related to exercise capacity, its potential prognostic value deserves investigation. In this study, the link between known prognostic factors and SctO_2_ during both rest and peak exercise were explored.

Methodologically, SctO_2_ was measured at the bilateral frontal region during the maximal incremental cardiopulmonary exercise test (CPET), in patients with systolic HFrEF, uni-hemisphere stroke, and healthy participants. Plasma level of brain natriuretic peptide (BNP) and complete blood cell count were collected in HFrEF and stroke groups. Physical parameters that determine SctO_2_ during rest and exercise were investigated. We hypothesized that SctO_2_ at rest and exercise have a close relationship with $\dot{V}O_{2peak}$ and are correlated with some known prognostic factors.

## Materials and methods

### Participants

This is a case-controlled cross-sectional design. Thirty-four stable HFrEF patients, 26 first-time uni-hemisphere ischemic stroke patients with hemiparesis, and 17 healthy controls were enrolled by convenience sampling in Linkou Chang Gung Memorial Hospital, a tertiary medical center. The enrolled stroke patients were at least 3 months post onset. All patients with HFrEF had a left ventricular ejection fraction ≤ 40% and a disease duration ≥ 3 months. Healthy controls were recruited by convenience sampling. Most of them were colleagues or caregivers of the hospitalized patients. The exclusion criteria included contraindications to stress exercise testing [12], or inability to ride a bike due to musculoskeletal problems or neurologic deficits including muscle strength less than 4 based on Medical Research Council scale. Patients with HFrEF with moderate to severe carotid artery stenosis or diseases that might affect ventilation, such as chronic obstructive pulmonary disease and pulmonary hypertension, were also excluded. In the stroke group, those with ventilation disorders were also excluded. In the healthy control group, those with any cardiovascular or respiratory diseases in the medical record were excluded. Written informed consent was obtained from every subject before the experiment. The study protocol was performed in accordance with the Declaration of Helsinki and approved by the ethics committee in Chang Gung Memorial Hospital, Linkou, Taiwan. Venous blood was sampled in the morning to determine BNP and complete blood cell count in the HFrEF and stroke groups.

### Cardiopulmonary exercise testing

All participants received symptom-limited incremental CPET with upright position on a calibrated bicycle ergometer (Ergoselect 150P, Germany). CPET started with 2 min of rest and 1 min warm-up at a work rate of 10 W followed by a ramp increase of 10 W/min until exhaustion. Heart rate was calculated from the R-R interval recorded on a 12-lead electrocardiogram. Blood pressure was measured automatically every 2 min (Tango, SunTech Medical, UK). Gas analysis was measured breath by breath using a microprocessor-controlled system (MasterScreen CPX, Cardinal-health Germany). The $\dot{V}O_{2peak}$ was achieved as the examinee failed to keep the cadence above 50/min despite strong encouragement. Mean arterial pressure (MAP) was calculated by the following equation: MAP = [(2 x diastolic) + systolic] / 3. Arterial oxygen saturation was measured by finger pulse oximetry (model 9500, Nonin Onyx, Plymouth, Minnesota).

### Ventilatory efficiency

Ventilation and carbon dioxide consumption ($\dot{V}CO_{2}$) responses, acquired from the initiation of exercise to peak values, were used to calculate the minute ventilation (V_E_)- $\dot{V}CO_{2}$ slope using least-squares linear regression (y = mx + b, m = slope), where a more horizontal slope suggests better ventilation efficiency [13]. The oxygen uptake efficiency slope (OUES) was derived from the slope of a natural logarithm plot of V_E_ vs. $\dot{V}O_{2}$. The OUES is an estimation of the efficiency of ventilation with respect to $\dot{V}O_{2}$, with greater slope indicating higher oxygen uptake efficiency [14].

### Cerebral oximetry

SctO_2_ was monitored using the FORE-SIGHT system (CAS Medical Systems, Inc., Branford, CT). The scanning frequency was 100 Hz and was averaged in one second for the value at rest and peak exercise. The SctO_2_ value at rest was picked up when it reached a stable baseline during two-minute pretest before the exercise started as sitting upright on the cycle ergometry. The FORE-SIGHT device is a spatially resolved near-infrared cerebral oximeter that measures the absolute value of SctO_2_. Four continuous near-infrared (bandwidth < 1 nm) wave-length (690 nm, 780 nm, 805 nm, and 850 nm) of lights penetrated the brain from the prism of sensors (3.625” x 1.5”). Reflected light was then sampled by detectors on the sensor. Four wave-lengths were employed to enhance measurement accuracy of oxyhemoglobin and deoxyhemoglobin levels by compensating for wavelength-dependent scattering losses and by eliminating interference from other background light absorbers (such as skin pigmentation and fluid) [15, 16]. Both resting and peak SctO_2_ were measured in the upright sitting posture on the stationary bike. Sensors were placed on the forehead bilaterally. The average value from bilateral forehead was analyzed along with the data from each side. They were used to represent tissue oxygenation at the frontal lobe. The validity has been established and approved by FDA in monitoring during cardiovascular surgery [16]. It has also been applied to measure cerebral oxygenation during exercise in patients with HFrEF [9, 17–19].

### Echocardiography

Echocardiography was performed in HFrEF and stroke groups by a cardiologist using Vivid E90 (GE Healthcare, Milwaukee, WI) equipped with a 2.5-MHz transducer. Left ventricular ejection fraction, left ventricular end-diastolic, and end-systolic volumes were quantitated by M-mode and two-dimensional methods [20].

### Statistical analysis

Statistical Package for the Social Sciences (SPSS) version 22.0 (SPSS, Inc., Chicago, IL, USA) was used to analyze the data. Continuous data were expressed as mean ± standard deviation. Three-group comparison was performed by ANOVA with Scheffe post hoc test in the continuous variables, and Chi-Squared test in the categorical ones. SctO_2_ during rest and peak exercise in the three groups were compared by two-way repeated measure ANOVA with Scheffe post hoc test. Chi-Squared test was also applied to analyze the asymmetric pattern of SctO_2_. Pearson correlation was used to analyze SctO_2_ versus other physical parameters, including Hb, BNP, and all the cardio-respiratory parameters listed in Table 1. The four variables with the highest correlation coefficients (all p < 0.05) were initially put into the linear stepwise univariate regression model to select the major variables associated with SctO_2_. Delta R-squared value was calculated to determine the change in R-squared value as adding another variable into the model. All probability values were two-tailed and the significance threshold was set at 0.05.

**Table 1:** Cardio-respiratory parameters in the incremental stress exercise testing.

		HFrEF (n = 34)	Stroke (n = 26)	Control (n = 17)	P value
**Cardiac parameters**					
HR_peak_	beats/min	124±19†	133±19‡	151±20	<0.001
SBP_peak_	mmHg	138±28†*	172.6±23	192±18	<0.001
MAP_rest_	mmHg	88±15†*	99±13	103±13	0.001
MAP_peak_	mmHg	98±16†*	116±14	126±8	<0.001
**Respiratory parameters**					
BF_peak_	breaths/min	30±8	34±11	37±6	0.083
V_Epeak_	L/min	40±11†	41.2±12.5‡	56.5±15.1	<0.001
V_tpeak_	L/min	1.4±0.5	1.3±0.4	1.5±0.3	0.29
$\dot{V}O_{2peak}$	ml/min/kg	13.4±5.7†	16±4‡	20.2±4.5	0.001
$\dot{V}CO_{2peak}$	ml/min/kg	15.8±6.5†	19±5.7‡	23.3±9.3	0.009
RER_peak_	-	1.26±0.58	1.18±0.14	1.14±0.34	0.659
$V_{E}/\dot{V}O_{2}$ nadir	-	29±5.8†	26.4±5	24.8±2.5	0.032
$V_{E}‑\dot{V}CO_{2}$ slope	-	34.3±6.5†*	29.7±4.2	26.6±1.7	<0.001
P_ET_O_2peak_	mmHg	119.4±7†*	115.3±4	113.7±4.5	0.003
P_ET_CO_2rest_	mmHg	30.5±7.7	33.9±4.5	37±2.2	0.003
OUES	-	460±125†*	591±176‡	784±195	<0.001

Values are means ± SD; HFrEF, heart failure with reduced ejection fraction; Peak, peak exercise; HR, heart rate; SBP, systolic blood pressure; MAP, mean arterial pressure; BF, breathing frequency; V_E_, minute ventilation; V_t_, tidal volume; VO_2_, O_2_ consumption; $\dot{V}CO_{2}$, CO_2_ production; RER, respiratory exchange ratio; P_ET_O_2_ and P_ET_CO_2_, end-tidal partial pressures of O_2_ and CO_2_; OUES, oxygen uptake efficiency slope

†: p < 0.05, HFrEF vs. control

‡: p < 0.05, stroke vs. control

*: p < 0.05, HFrEF vs. stroke; ANOVA with Scheffe post hoc test

## Results

No significant differences in age, gender, height, weight, and body mass index were shown between the three groups (Table 2). Plasma BNP and Hb were 974±966 (pg/mL) and 13.1±2.6 (g/dL) in the HFrEF group; 469±393 (pg/mL) and 14.6±1.7 (g/dL) in the stroke group. $\dot{V}O_{2peak}$ and OUES were 13.4±5.7 (ml/min/kg) and 460±125 in the HFrEF group; 16±4 (ml/min/kg) and 591±176 in the stroke group; 20.2±4.5 and 784±195 in the control group. Mean arterial pressure (MAP) at rest and peak were 88±15 (mmHg) and 98±16 (mmHg) in the HFrEF group; 99±13 (mmHg) and 116±14 (mmHg) in the stroke group; 103±13 (mmHg) and 126±8 (mmHg) in the control group (Table 1).

**Table 2:** Demographic and clinical characteristics.

		HFrEF (n = 34)	Stroke (n = 26)	Control (n = 17)	P value
Gender	n (M/F)	31/3	20/6	15/2	0.316
Age	year	56±13	58±11	56±14	0.684
Height	cm	166.2±7.8	162.4±8.6	164.8±7.3	0.183
Weight	kg	67.2±12.5	66.3±10.4	67.0±13.9	0.959
BMI	kg/meter^2^	24.2±3.8	25.1±2.6	24.5±3.5	0.636
Comorbidities					
Hypertension	n (%)	13 (38)	17 (61)	-	0.126
Hyperlipidemia	n (%)	5 (15)	4 (14)	-	1.000
Smoking	n (%)	4 (12)	2 (7)	-	0.678
Diabetes mellitus	n (%)	11 (32)	5 (18)	-	0.245
Sleep apnea	n (%)	0 (0)	2 (7)	-	0.207
Coronary artery disease	n (%)	4 (12)	1 (4)	-	0.363
Atrial fibrillation	n (%)	4 (12)	2 (7)	-	0.678
Overweight	n (%)	3 (9)	0 (0)	-	0.243
Medication					
ACEI	n (%)	2 (6)	1 (4)	-	1.000
ARB	n (%)	7 (20)	5 (18)	-	1.000
B-blocker	n (%)	17 (50)*	6 (21)	-	<0.05
Ca^++^ channel blocker	n (%)	8 (24)*	15 (54)	-	<0.05
Diuretics	n (%)	23 (68)*	5 (18)	-	<0.05
Nitrates	n (%)	5 (15)	8 (29)	-	0.227
Digoxin	n (%)	12 (35)*	3 (11)	-	<0.05
Anti-arrhythmic		1 (3)	1 (4)	-	1.000
Echocardiography					
LVEF	%	32±14*	64±10	-	<0.05
LVEDD	mm	60±10.1*	48±7	-	<0.05
LVESD	mm	47±13*	31±6	-	<0.05
Hb	g/dL	13.1±2.6*	14.6±1.7	-	<0.05
BNP	pg/mL	549 (232–1605)*	329 (182–1063)	-	<0.05
Hemisphere lesion	right: left		11:15		

Values except BNP are means ± SD; BNP is presented as median (25th to75th percentile)

Overweight is defined as BMI greater than or equal to 25

HFrEF, heart failure with reduced ejection fraction; M, male; F, female; BMI, body mass index; COPD, chronic obstructive pulmonary disease; ACEI, angiotensin-converting-enzyme inhibitor; ARB, Angiotensin II receptor blocker; LVEF, left ventricular ejection fraction; LVEDD, left ventricular end diastolic diameter; LVESD, left ventricular end systolic diameter; Hb, hemoglobin; BNP, B-type natriuretic peptide.

*: p < 0.05, HFrEF vs. stroke; independent t-test for continuous variables and Chi-Squared test for categorical ones

**Fig 1:**
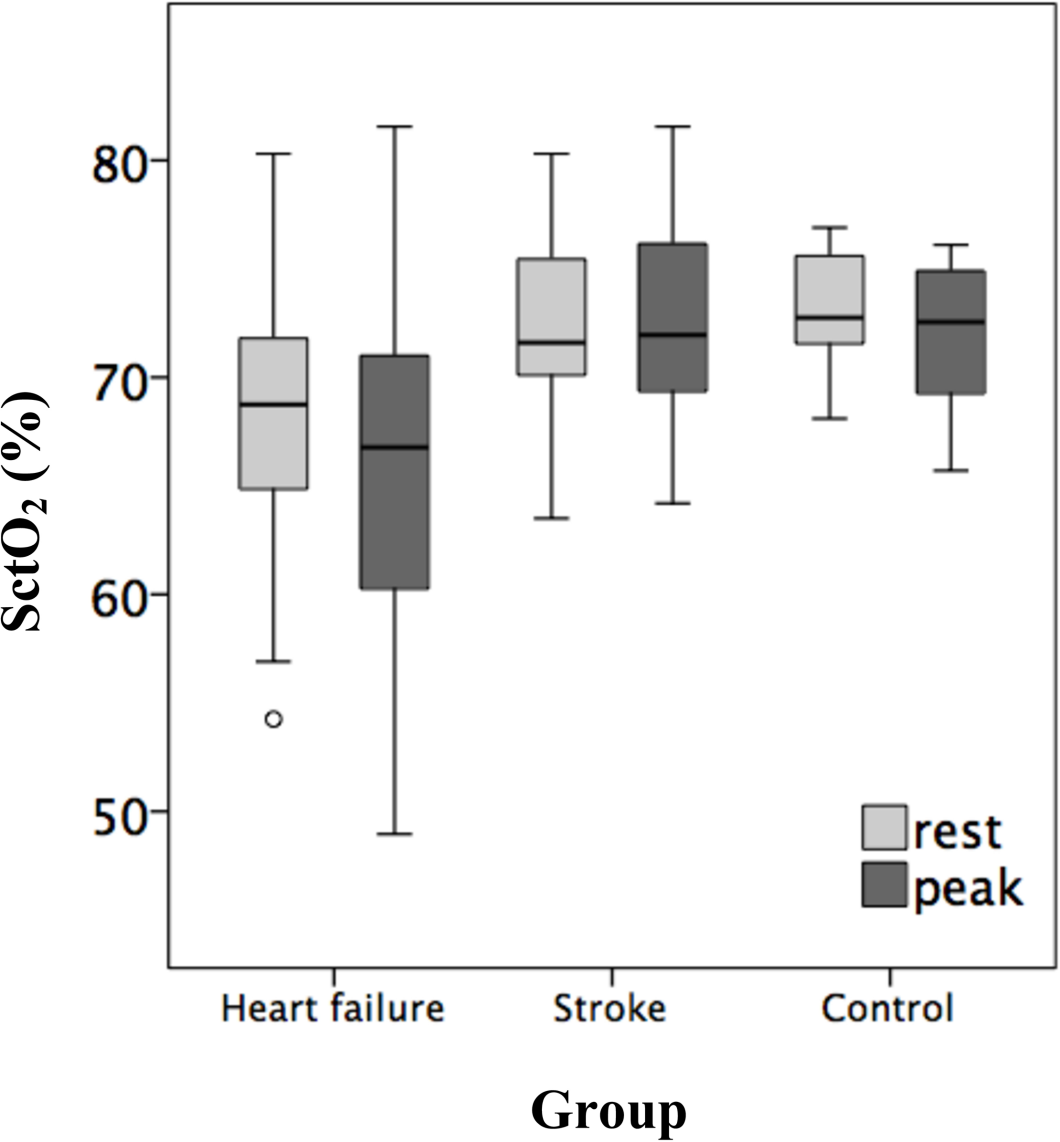
Boxplots of SctO_2_ during rest and peak exercise among the three groups.

Oxygen saturation of cerebral tissue (rest; peak) were significantly lower in the HFrEF (66.3±13.3%; 63.4±13.8%) than that in the stroke (72.1±4.2%; 72.7±4.5%) and control (73.1±2.8%; 72±3.2%) groups as revealed by two-way repeated measure ANOVA, while those in the stroke group were close to the healthy control. Moreover, in the HFrEF group, SctO_2_ decreased significantly at peak exercise, a phenomenon not observed in the stroke or healthy control groups (Fig 1, Table 3). Additionally, all the arterial oxygen saturation among three groups were 98~100% during both rest and peak exercise.

**Table 3:** Cerebral tissue oxygen saturation in incremental exercise testing.

		HFrEF (n = 34)		Stroke (n = 26)		Control (n = 17)		P value
		Rest	Peak	Rest	Peak	Rest	Peak	
SctO_2_	%	66.3±13.3	63.4±13.8†*	72.1±4.2	72.7±4.5	73.1±2.8	72±3.2	<0.001
L't SctO_2_	%	68.1±5.9	65.5±7.8†*	71.8±4.1	72.5±4.7	72.9±2.6	71.6±2.8	<0.001
R't SctO_2_	%	68.6±6.1	65.2±7.8†*	72.6±5.1	73.3±7.3	73.3±3.1	72.6±3.6	<0.001

Values are means ± SD; HFrEF, heart failure with reduced ejection fraction; Peak, peak exercise

L’t, Left; R’t, Right; SctO_2_, cerebral tissue oxygen saturation

†: p < 0.05, HFrEF vs. control

*: p < 0.05, HFrEF vs. stroke; repeated measure ANOVA with Scheffe post hoc test

**Fig 2:**
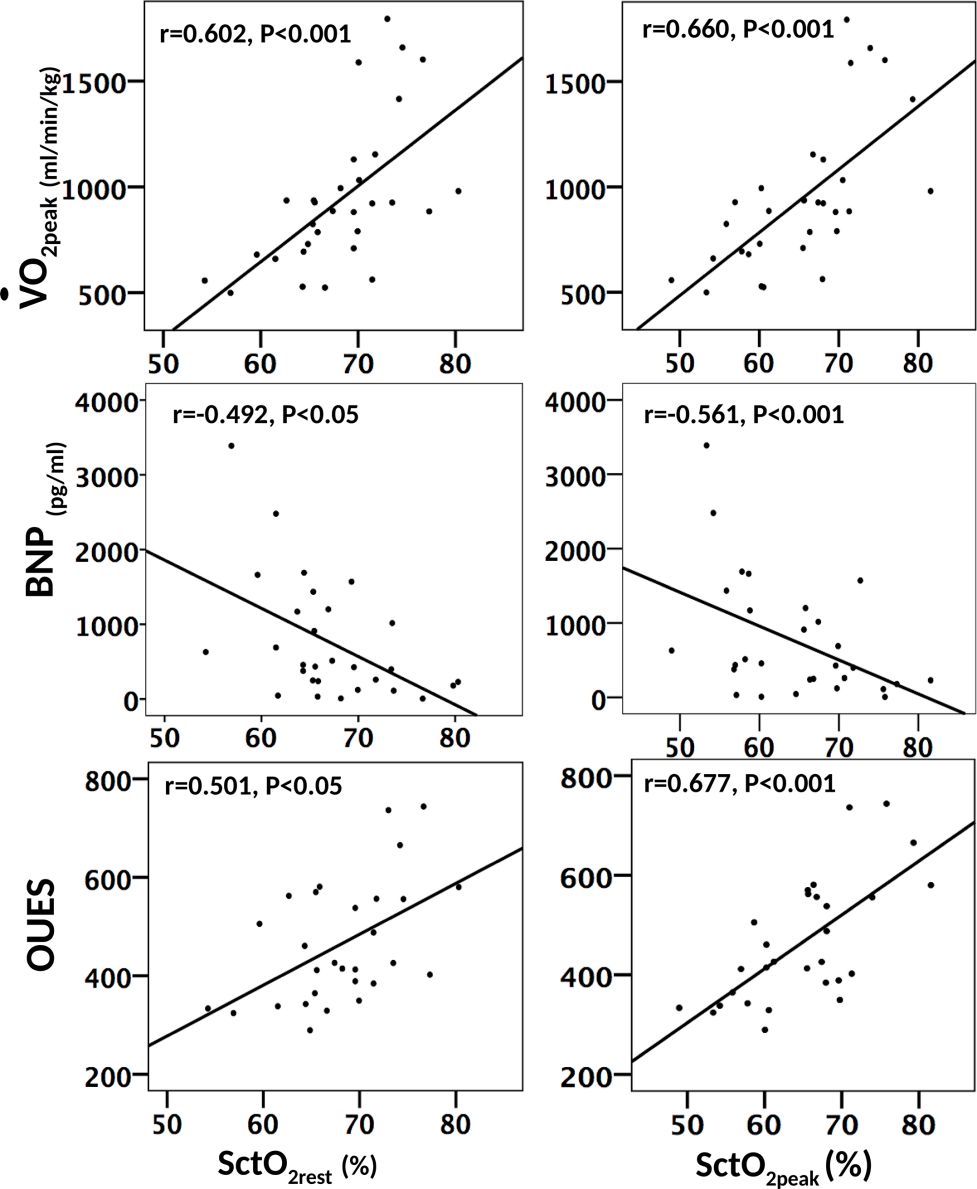
Scatter plots of SctO_2rest_ and SctO_2peak_ against $\dot{V}O_{2\mathrm{peak}}$, BNP and OUES.

Pearson correlation was performed between SctO_2_ and cardio-respiratory and hematologic parameters. In the HFrEF group, SctO_2rest_ and SctO_2peak_ were positively correlated with $\dot{V}O_{2peak}$ (*r* = 0.602, *p* < 0.001; *r* = 0.660, *p* < 0.001) and OUES (*r* = 0.501, *p* < 0.05; *r* = 0.677, p < 0.001) and negatively correlated with BNP (*r* = -0.492, *p* < 0.05; *r* = -0.561, *p* < 0.001) (Fig 2). On the other hand, the change in SctO_2_ from rest to peak (-1.4 ± 3.3%) was not correlated with the investigated prognostic factors, including $\dot{V}O_{2peak}$ (*r* = -0.430), OUES (*r* = -0.362), BNP (*r* = -0.230), $V_{E}‑\dot{V}CO_{2}$ slope (*r* = -0.025), P_ET_CO_2rest_ (*r* = -0.177) and SBP_peak_ (*r* = -0.263). Additionally, no significant correlation was found between SctO_2_ and cardio-respiratory or hematologic parameters in the stroke and control groups.

Linear stepwise univariate regression analysis was employed to identify the major physiologic determinants of SctO_2rest_ and SctO_2peak_ in patients with HFrEF. $\dot{V}O_{2peak}$ (*r* = 0.602), MAP_rest_ (*r* = 0.597), partial pressure of end-tidal carbon dioxide at rest (P_ET_CO_2rest_) (*r* = 0.586), Hb (*r* = 0.51), and were entered into the model of SctO_2rest_. OUES (*r* = 0.677), $\dot{V}O_{2peak}$ (*r* = 0.66), peak carbon dioxide production ($\dot{V}CO_{2peak}$) (*r* = 0.651), and BNP (*r* = -0.561) were entered into the model of SctO_2peak_ (S1 Table). It revealed that SctO_2rest_ was determined by P_ET_CO_2rest_, Hb, and MAP_rest_ (R = 0.681, *p* < 0.05), while SctO_2peak_ was by VCO_2peak_ (R = 0.653, *p* < 0.05) (Tables 4 and 5). It is worth mentioning that Hb is also significantly correlated with SctO_2peak_ (*r* = 0.39). However, it does not increase the predictive power significantly when it was entered after $\dot{V}CO_{2peak}$ (p-value of ß was 0.052).

**Table 4:** Linear regression modeling of SctO_2rest_ in HFrEF group.

	ß	t	*P(*ß*)*	R	ΔR^2^	F
Model 1				0.593	0.352	14.679*
**P**_**ET**_**CO**_**2rest**_	0.593	3.831	0.001			
Model 2				0.639	0.056	19.257*
**P**_**ET**_**CO**_**2rest**_	0.552	3.757	0.001			
**Hb**	0.314	2.140	0.042			
Model 3				0.681	0.056	25.009*
**P**_**ET**_**CO**_**2rest**_	0.517	3.804	0.001			
**Hb**	0.331	2.451	0.022			
**MAP**_**rest**_	0.323	2.398	0.024			

P_ET_CO_2_, the end-tidal partial pressures of CO_2_; Hb, hemoglobin; MAP, mean arterial pressure

* p < 0.05; the p-value indicates the overall significance of the linear regression model

P(ß): p-value for ß; R and ΔR^2^ are adjusted values

**Table 5:** Linear regression modeling of SctO_2peak_ in HFrEF group.

	ß	t	*P(*ß*)*	R	ΔR^2^	F
Model 1				0.653	0.426	13.624*
$\dot{V}CO_{2peak}$	0.678	3.691	0.002			

$\dot{V}CO_{2peak}$: peak CO_2_ production

* p < 0.05; the p-value indicates the overall significance of the linear regression model

P(ß): p-value for ß; R and ΔR^2^ are adjusted values

The experimental finding of bilateral comparison of SctO_2_ in the HFrEF vs. control groups and its related discussion are appended in the supporting information (S1 Supporting information).

## Discussion

To the best of our knowledge, the current investigation is the first to determine the potential prognostic value of SctO_2_ and its possible physiologic basis in patients with HFrEF. Significant experimental findings were as follows: I. SctO_2_ of the HFrEF group were significantly decreased compared with healthy control and stroke groups. II. Patients with HFrEF not only had diminished cerebral oxygenation at rest, but also showed further reduced oxygenation during incremental exercise testing, which was not observed in the stroke and healthy groups. III. Importantly, both SctO_2rest_ and SctO_2peak_ of the HFrEF group (especially SctO_2peak_) were correlated with $\dot{V}O_{2peak}$, BNP, and OUES in moderate degree, which are well-established prognostic markers. IV. In the HFrEF cohort, linear regression analysis showed that SctO_2rest_ was primarily determined by P_ET_CO_2rest_, Hb and MAP_rest_, while SctO_2peak_ was affected primarily by $\dot{V}CO_{2peak}$. V. The numerical value of SctO_2_ did not differ between the stroke and healthy control groups. Its association with other investigated biomarkers was not obvious in these two groups either.

### Low cerebral oxygenation in patients with HFrEF

CBF is reduced in patients with mild to severe HFrEF even in resting state, as confirmed through ^133^XE radionuclide angiography injection method, and transcranial and extracranial Doppler ultrasonography [21–23]. During dynamic exercise, cerebral perfusion can be sacrificed to active skeletal muscle in patients with HFrEF. Hellstrom et al. demonstrated that healthy subjects had a 20% increase in mean middle cerebral artery blood velocity (MCA V_mean_) when performing one-legged exercise and this increase was maintained when performing two-legged exercise. However, in patients with HFrEF, MCA V_mean_ was not increased during one-legged exercise and was significantly decreased during two-legged exercise [24]. For stroke patients, Robertson et al. reported that regional CBF was reduced after low-intensity exercise but increased after moderate-intensity exercise [25]. Similarly, the present investigation found that SctO_2_ was lower in patients with HFrEF during resting state and decreased significantly from rest to peak exercise only in the HFrEF group. It is very possible that cerebral autoregulation fails to compensate CBF in face of low systemic BP commonly seen in HFrEF patients, leading to reduced SctO_2_ [26]_._ The reason why our data did not demonstrate cerebral oxygenation reduction in the stroke group could be that only patients with mild severity of stroke were recruited. Their muscle strength in the hemiparetic limbs were 4 or 5- in manual muscle testing. Thus, the data pattern was similar in the stroke and healthy control groups. Meanwhile, the potential influence of medications should be considered. Calcium channel blockers (CCB) elevate CBF[27], while beta-blockers attenuate the increase in cardiac output, CBF and cerebral oxygenation during exercise [28, 29]. In our study, significantly more patients took CCB in the stroke group, while more patients took beta-blockers in the HFrEF group. Although these medications may augment cerebral deoxygenation in HFrEF patients, the prescription was in line with the current treatment guidelines and reflects the real-world situation [30, 31].

### Potential prognostic value of SctO_2_ in HFrEF

Our data showed that in patients with HFrEF, both SctO_2rest_ and SctO_2peak_ (especially SctO_2peak_) were correlated with $\dot{V}O_{2peak}$, BNP, and OUES in moderate degree. BNP has been recognized as a prognostic and diagnostic factor for HFrEF and is used to assess the severity of HFrEF [32, 33]. $\dot{V}O_{2peak}$ provides objective assessment for cardio-pulmonary fitness and is one of the most significant short-term and long-term prognostic factors for patients with HFrEF [34, 35]. OUES measures oxygen uptake efficiency in relation to ventilation during an incremental exercise test and has been shown to be a prognostic marker, which is even more predictive than $\dot{V}O_{2peak}$ in some of the HFrEF studies [14, 36].

Koike et al. found that in patients with coronary artery disease, the change of cerebral oxyhemoglobin (O_2_Hb) from rest to peak exercise is prognostic for cardiovascular morbidity [37]. However, our results showed that the difference between SctO_2rest_ and SctO_2peak_ were not associated with the known prognostic factors investigated. Instead, it was the absolute value during peak or rest that might be related to prognosis. An explanation is that O_2_Hb and SctO_2_ are essentially different. Decrease of O_2_Hb from rest to peak exercise may result from the reduction of CBF or increased oxygen consumption of the tissue. However, SctO_2_ is decreased only when O_2_Hb has a relatively larger reduction than deoxyhemoglobin [24]. Probably, it is the reduction of CBF that is primarily associated with prognosis.

### Determinants of SctO_2rest_ in HFrEF: P_ET_CO_2rest_, Hb, and MAP_rest_

The linear stepwise regression model indicated that SctO_2rest_ of the HFrEF group was primarily determined by P_ET_CO_2rest_, Hb, and MAP_rest_. The explanatory power was 46% (R^2^ = 0.464) in this model. P_ET_CO_2_ is very close to partial pressure of arterial carbon dioxide (PaCO_2_) during rest since those with ventilation problems were excluded in the HFrEF group [38–40]. Therefore, our experimental findings of P_ET_CO_2_ during rest may be interpreted as PaCO_2_. PaCO_2_ was previously demonstrated to be positively linearly correlated to CBF from 15 to 60 mmHg [41]. Our data of P_ET_CO_2_ in the HFrEF group and the control group were 30.5±7.7 and 37±2.2 mmHg, respectively. Therefore, the reduced P_ET_CO_2_ in the HFrEF group may affect SctO_2rest_ due to decreased CBF. In addition, low P_ET_CO_2_ is also a significant predictor of cardiac-related events in patients with HFrEF [42].

Cerebral oxygenation is also influenced by arterial oxygen concentration [43]. Anemia leads to a decreased oxygen supply to the cerebral tissue. It is also associated with an increased mortality rate in patients with HFrEF [44, 45]. In healthy subjects, the disadvantage of anemia is compensated by increased cardiac output, plasma volume, reduced systemic vascular resistance, and widened arteriovenous oxygen gradient. However, these compensatory mechanisms are impaired in patients with HFrEF [46, 47].

MAP also influences cerebral oxygenation based on our results. Herholz et al. adopted ^133^Xe clearance technique and demonstrated that the linear increase in CBF resulted not only from PaCO_2_ but also MAP [41]. Rifai et al. also found a positive correlation between SctO_2_ and MAP in patients with HFrEF at rest [48]. Higher MAP may be associated with higher cerebral perfusion [49]. As perfusion into the tissue vascular bed is increased, Hb density in the tissue increases and thereby, SctO_2_ is less reduced. Accordingly, MAP_rest_, plays a role in determining CBF and thereby SctO_2rest_. Meanwhile, MAP was inversely related to the total and cardiovascular mortality [50], which as well indicated potential prognostic value of SctO_2_. Nonetheless, MAP was not correlated with SctO_2peak_. Previous investigations confirmed that CBF does not increase or even decline at peak exercise even though MAP rises in patients with HFrEF [9, 51]. In other words, MAP does not reflect CBF during exercise in patients with HFrEF. In summary, P_ET_CO_2rest_, Hb, and MAP_rest_ are important determinants of SctO_2rest_, as well as prognostic markers in patients with HFrEF.

### Determinants of SctO_2peak_ in HFrEF: $\dot{V}CO_{2peak}$

The stepwise linear regression showed that the most major determinant of SctO_2peak_ in the HFrEF group is $\dot{V}CO_{2peak}$ (R = 0.653; R^2^ = 0.426). There are two explanations: first, $\dot{V}CO_{2peak}$ and $\dot{V}O_{2peak}$ have high collinearity. Therefore, low $\dot{V}CO_{2peak}$ suggests low oxygen delivery to the peripheral tissue, including brain and thus leads to decreased SctO_2_ [43]. Second, patients with HFrEF with lower $\dot{V}CO_{2peak}$ (thus, lower $\dot{V}O_{2peak}$) tend to have lower PaCO_2_ due to ventilation-perfusion mismatch [43], which results in cerebral vasoconstriction [52]. This could explain why SctO_2peak_ in the HFrEF group is primarily determined by $\dot{V}CO_{2peak}$ rather than $\dot{V}O_{2peak}$.

### SctO_2_ in stroke

The numerical value of SctO_2_ in the stroke and healthy control groups were not different based on the present data. Also, it was not associated with the investigated known prognostic factors. It can be explained by the mild severity in the included stroke patients. Their muscle strength in the hemiparetic limbs were 4 or 5- in manual muscle testing. In consideration of ergometer-riding being the modality of exercise testing, those with poorer muscle strength were not adequate to be included because maximal effort cannot be reached due to the neurologic deficits. In Table 3, the respiratory exchange ratio of the stroke group was not different from that of the control group (1.18±0.14 vs. 1.14±0.34), indicating that maximal exertion was nearly approached in the stroke group. The data showed that although patients with mild ischemic stroke were less fit (lower VO_2peak_, HR_peak_ and V_Epeak_) than the control group, SctO_2_ during rest or peak were not lower. Further study on stroke patients with higher severity and significant cerebrovascular stenosis is needed.

### Limitations of NIRS cerebral oximetry

Some limitations concerning NIRS cerebral oximetry need to be taken into consideration. First, SctO_2_ was measured without differentiating vascular bed as being arterial, capillary, or venous. Since it is estimated that more than 70% of the Hb in the brain is in venous bed, the measured SctO_2_ may reflect larger proportion of venous saturation [53]. Second, extracranial contamination and melanin may absorb light and thus attenuate the signal, though all the participants are Asian. [54, 55].

### Study limitation

A longitudinal study is needed to confirm the prognostic value of SctO_2_. Moreover, P_ET_CO_2_ rather than PaCO_2_ was measured in the current study though patients with ventilation disorder were already excluded. However, in patients with advanced HFrEF, a certain degree of increased dead space ventilation may be present; thus, PaCO_2_ might be slightly higher than P_ET_CO_2_, even at rest [43]. Additionally, lack of direct CO and CBF measurement and absence of echocardiographic data, Hb, and BNP in the control group limit precise interpretation and analysis. Also, it may influence the SctO_2_ value in the stroke group to have the NIRS sensors placed on the fixed positions regardless of whether the infarction area was right underneath the sensor.

## Conclusion

Cerebral oxygenation was reduced in patients with HFrEF compared with healthy controls. Moreover, cerebral oxygenation, especially at peak exercise, is correlated with $\dot{V}O_{2peak}$, BNP, and OUES, which are well-recognized prognostic factors. Cerebral oxygenation during rest is determined mainly by P_ET_CO_2_, Hb, and MAP_rest_, while at peak exercise, is primarily affected by $\dot{V}CO_{2peak}$ in patients with HFrEF. These findings provided the physiologic basis of cerebral oxygenation and its potential prognostic value in patients with HFrEF, and may have clinical value in the future.

## Supporting information

S1 Table Pearson correlation coefficients between cerebral tissue oxygen saturation and cardio-respiratory variables. (DOCX)

S1 Supporting information Comparison of bilateral cerebral tissue oxygen saturation at peak exercise in HFrEF vs. control and stroke vs. control groups. (DOCX)

## Abbreviations

BNP: brain natriuretic peptide
CBF: cerebral blood flow
CO: cardiac output
CPET: cardiopulmonary exercise test
Hb: hemoglobin
HFrEF: heart failure with reduced ejection fraction
MAP: mean arterial pressure
MCA V_mean_: mean middle cerebral artery blood velocity
O_2_Hb: oxyhemoglobin
OUES: oxygen uptake efficiency slope
PaCO_2_: partial pressure of arterial carbon dioxide
P_ET_CO_2_: partial pressure of end-tidal carbon dioxide
SctO_2_: cerebral tissue oxygen saturation
$\dot{V}CO_{2}$: carbon dioxide production
V_E_: minute ventilation
$\dot{V}O_{2}$: oxygen consumption
