# Supplementary material for: Cerebral desaturation in heart failure: Potential prognostic value and physiologic basis
Source: PLoS One. 2018 Apr 24;13(4):e0196299. doi: 10.1371/journal.pone.0196299 (PMC5916527; doi:10.1371/journal.pone.0196299)
Supplement: S1 Table — (DOCX) [file pone.0196299.s001.docx]

**S1 Table. Pearson correlation coefficients between cerebral tissue oxygen saturation and cardio-respiratory variables**

|  | Hb | BNP | HR_peak_ | SBP_peak_ | MAP_rest_ | MAP_peak_ | BF_peak_ | V_Epeak_ | V_tpeak_ | VO_2peak_ | VCO_2peak_ | VE/VO_2_ nadir | VE-VCO_2_ slope | P_ET_CO_2rest_ | OUES |
| --- | --- | --- | --- | --- | --- | --- | --- | --- | --- | --- | --- | --- | --- | --- | --- |
| SctO_2rest_  0.510* | | -0.492* | 0.252 | 0.119 | 0.597* | 0.200 | -0.295 | 0.474 | 0.426 | 0.602* | 0.418 | -0.478 | -0.448 | 0.586* | 0.501* |
| SctO_2peak_  0.390* | | -0.561* | 0.140 | 0.214 | 0.351 | 0.293 | -0.353 | 0.384 | 0.521* | 0.660* | 0.651* | -0.555* | -0.531* | 0.330 | 0.677* |

SctO_2_, cerebral tissue oxygen saturation; Hb, hemoglobin; BNP: brain natriuretic peptide; Peak, peak exercise; SBP, systolic blood pressure; MAP, mean arterial pressure; BF, breathing frequency; V_E_, minute ventilation; V_t_, tidal volume; VO_2_, O_2_ consumption; VCO_2_, CO_2_ production; RER, respiratory exchange ratio; P_ET_O_2_ and P_ET_CO_2_, end-tidal partial pressures of O_2_ and CO_2_; OUES, oxygen uptake efficiency slope

*: p < 0.05
