# Supplementary material for: Cerebral desaturation in heart failure: Potential prognostic value and physiologic basis
Source: PLoS One. 2018 Apr 24;13(4):e0196299. doi: 10.1371/journal.pone.0196299 (PMC5916527; doi:10.1371/journal.pone.0196299)
Supplement: S1 Supporting information — (DOCX) [file pone.0196299.s002.docx]

**S1 Supporting information.**

**Comparison of bilateral cerebral tissue oxygen saturation at peak exercise in HFrEF vs. control and stroke vs. control groups**

|  | | **HFrEF** | **control** | **total** | **stroke** | **control** | **total** |
| --- | --- | --- | --- | --- | --- | --- | --- |
| **SctO_2_ lower side**  (number) | **Rt** | 21 | 3 | 24 | 9 | 3 | 12 |
|  | **Lt** | 13 | 14 | 27 | 17 | 14 | 31 |
|  | **total** | 34 | 17 | 51 | 26 | 17 | 43 |

Rt, right; Lt, left

*P<0.05, Chi-Squared test: HFrEF vs. control

**Result**

In both the stroke and control groups, more subjects had lower SctO_2rest_ and SctO_2peak_ over the left hemisphere than the right, while this proportion was on the opposite in HFrEF group. SctO_2peak_ in the HFrEF group significantly lost the asymmetric pattern (left hemisphere lower than right side) as observed in the control group (*p* = 0.003) (S2). Additionally, uni-hemisphere vascular lesion in the stroke group did not change this asymmetric pattern.

**Discussion**

Hemispheric lateralization has been reported in cerebral functions including motor control and language [1]. Lateralization of brain function may result in unequal oxygen demand and consumption, thus leading to lower SctO_2rest_ in the left hemisphere. Loss of the asymmetric pattern of brain oxygenation can be caused by impaired cerebral autoregulation during exercise in patients with HFrEF [2]. Theoretically, asymmetry in cerebral oxygenation may be attributed to vascular lesions in the cerebral circulation. However, our data showed that the side of uni-hemisphere vascular lesion in the stroke group did not change this pattern compared to healthy controls. Whether this observation has physiologic or clinical significance needs further investigation.

**Reference**

1. Duboc V, Dufourcq P, Blader P, Roussigne M. Asymmetry of the Brain: Development and Implications. Annual review of genetics. 2015;49:647-72. Epub 2015/10/08. doi: 10.1146/annurev-genet-112414-055322. PubMed PMID: 26442849.

2. Paulson OB, Strandgaard S, Edvinsson L. Cerebral autoregulation. Cerebrovascular and brain metabolism reviews. 1990;2(2):161-92. Epub 1990/01/01. PubMed PMID: 2201348.
